# Supplementary material for: Mechanisms governing the pioneering and redistribution capabilities of the non-classical pioneer PU.1
Source: Nat Commun. 2020 Jan 21;11:402. doi: 10.1038/s41467-019-13960-2 (PMC6972792; doi:10.1038/s41467-019-13960-2)
Supplement: Supplementary file 7 — Source data [file 41467_2019_13960_MOESM7_ESM.zip › Source_Data/FigureS3/FigureS3J_MotifScanOutput/homerResults/motif36.similar.html]

motif36

## Information for motif36

C
A
T
G
A
T
C
G
A
T
G
C
A
G
T
C
C
G
T
A
A
C
T
G
T
A
C
G
A
T
G
C
A
G
T
C
C
G
A
T
A
C
T
G
T
A
C
G
T
A
G
C
  
Reverse Opposite:  

A
T
C
G
A
T
G
C
A
G
T
C
G
C
T
A
A
C
T
G
A
T
C
G
A
T
G
C
A
G
T
C
G
C
A
T
A
C
T
G
T
A
C
G
A
T
G
C
G
T
A
C
  

|  |  |
| --- | --- |
| p-value: | 1e-37 |
| log p-value: | -8.595e+01 |
| Information Content per bp: | 1.669 |
| Number of Target Sequences with motif | 1255.0 |
| Percentage of Target Sequences with motif | 9.51% |
| Number of Background Sequences with motif | 2219.3 |
| Percentage of Background Sequences with motif | 6.08% |
| Average Position of motif in Targets | 569.9 +/- 590.7bp |
| Average Position of motif in Background | 316.3 +/- 200.9bp |
| Strand Bias (log2 ratio + to - strand density) | -0.1 |
| Multiplicity (# of sites on avg that occur together) | 1.25 |
| Motif File: | file (matrix) reverse opposite |

### Similar de novo motifs found

|  |  |  |  |  |  |  |  |
| --- | --- | --- | --- | --- | --- | --- | --- |
| Rank | Match Score | Redundant Motif | P-value | log P-value | % of Targets | % of Background | Motif file |
| 1 | 0.861 | T A G C C T G A C T A G T A C G A T G C A G T C C G A T T A C G A T G C A G T C G A T C | 1e-32 | -74.155989 | 11.96% | 8.36% | motif file (matrix) |
| 2 | 0.885 | A T C G T A G C G T A C C G T A C T A G T A C G T A G C G T A C G C T A C T A G A T C G T A G C G T A C G T C A | 1e-31 | -71.634871 | 11.06% | 7.66% | motif file (matrix) |
